# Lifetime Prevalence of Self-Reported Work-Related Health Problems Among U.S. Workers — United States, 2018

**DOI:** 10.15585/mmwr.mm6913a1

**Published:** 2020-04-03

**Authors:** Hannah Free, Matthew R. Groenewold, Sara E. Luckhaupt

**Affiliations:** 1 Division of Field Studies and Engineering, National Institute for Occupational Safety and Health, CDC.

Approximately 2.8 million nonfatal workplace illnesses and injuries were reported in the United States in 2018 (*1*). Current surveillance methods might underestimate the prevalence of occupational injuries and illnesses (*2*,*3*). One way to obtain more information on occupational morbidity is to assess workers’ perceptions about whether they have ever experienced health problems related to work (*4*). Occupational exposures might directly cause, contribute to, exacerbate, or predispose workers to various health problems (work-related health problems). CDC’s National Institute for Occupational Safety and Health estimated the lifetime prevalence of self-reported, work-related health problems for the currently employed population overall and stratified by various demographic and job characteristics using data from the 2018 version of the SummerStyles survey. Overall, 35.1% of employed respondents had ever experienced a work-related health problem (95% confidence interval [CI] = 33.0%–37.3%). The most commonly reported work-related health problem was back pain (19.4%, 95% CI = 17.6%–21.2%). Among industries, construction (48.6%, 95% CI = 36.54%–60.58%) had the highest prevalence of any work-related health problems. Workplace injury and illness prevention programs are needed to reduce the prevalence of work-related health problems, especially in higher-risk industries.

The SummerStyles survey is one in a series of annual, online surveys conducted by the communications firm Porter Novelli Public Services using panelists recruited using probability-based sampling methods. It has been conducted since 1995 and evaluates respondents’ beliefs about health topics including self-reported health problems (*5*). SummerStyles survey data have been demonstrated to be valid for reporting health outcomes when compared with the Behavioral Risk Factor Surveillance System (*5*,*6*).

In its 2018 survey, SummerStyles included questions about job characteristics of currently employed adults and whether respondents had experienced various types of work-related health problems. The survey was sent to 5,584 panelists; the response rate was 73.2%. The full survey sample included 4,088 adults aged ≥18 years. Work-related questions were only administered to adult respondents who described themselves as full-time paid employees, part-time paid employees, or self-employed, representing a sample of 2,425 for this analysis. Additional SummerStyles questions collect data on demographic characteristics including age, race, ethnicity, and education, as well as employment situation, industry sector, occupation category, and type of work arrangement.

Current workers who had ever experienced work-related health problems were identified by their response to the question “Have you ever experienced any of the following health problems related to any job you have ever held?” Respondents were asked to select all options that applied to them from a list of major categories of injuries and illnesses commonly related to work. This included 1) an injury that required medical treatment, 2) an injury that caused the respondent to miss work, 3) back pain, 4) other joint or muscle problem, 5) asthma or other lung condition, 6) hearing difficulty, 7) cancer, 8) mental health problem (e.g., depression), 9) skin condition, and 10) other health problem not listed. Respondents could also choose the option “no health problems related to work” or “I don’t know.” Point estimates and 95% CIs for the weighted* lifetime prevalence of any work-related health problem and specific types of work-related health problems among all workers were calculated. Prevalence ratios (PRs) were calculated to compare the prevalence of any work-related health problem across demographic and job characteristics. Analyses were performed using SAS statistical software (version 9.4; SAS Institute).

The overall lifetime prevalence of any work-related health problem was 35.1% (Table 1). The most commonly reported work-related health problem was back pain, reported by 19.4% of respondents; 14.7% of respondents reported a work-related injury that required medical treatment.

**TABLE 1:** Overall weighted* lifetime prevalence of work-related health problems — SummerStyles Survey, United States, 2018

Work-related health problem	Raw count (n = 2,425)^†^	Weighted % (95% CI)
Any work-related health problem	886	35.1 (33.0–37.3)
Back pain	488	19.4 (17.6–21.2)
Injury that required medical treatment	385	14.7 (13.2–16.3)
Injury that caused missed work	307	11.5 (10.1–12.9)
Other joint or muscle problem	286	10.9 (9.5–12.2)
Mental health problem (e.g., depression)	150	6.3 (5.1–7.4)
Other health problem not listed	66	2.9 (2.1–3.6)
Skin condition	61	2.5 (1.8–3.2)
Asthma or other lung condition	49	2.2 (1.4–2.9)
Hearing difficulty	59	1.8 (1.3–2.3)
Cancer	18	0.6 (0.32–0.9)

**Abbreviation**: CI = confidence interval.

* By gender, age, income, race, ethnicity, household size, education, U.S. Census region, and metro status, using U.S. Current Population Survey proportions.

^†^ Question responses were not mutually exclusive; therefore, totals do not sum to 2,425.

The prevalence of any work-related health problem did not vary significantly by sex; however, there was significant variation by age group, education, and race/ethnicity (Table 2). Respondents aged 55–64 years reported the highest prevalence of work-related health problems (41.3%), nearly twice that of persons aged 18–24 years (21.7%), and prevalences among all age groups except respondents aged ≥75 years were significantly higher than those of respondents aged 18–24 years. Non-Hispanic multiracial respondents had the highest prevalence of work-related health problems (49.1%). Prevalence among non-Hispanic blacks (39.9%) was also significantly higher compared with that of non-Hispanic other race respondents (28.2%). By educational attainment, prevalence was highest (39.2%) among respondents with less than a high school diploma and lowest (30.6%) among those with a bachelor’s degree or higher. The prevalence of any work-related health problem did not vary significantly by occupation, or work arrangement, but did vary significantly by industry and employment situation. Compared with the referent (Education) prevalence ratios were significantly higher for the Construction (PR = 1.6; 95% CI = 1.2%–2.2%), Public Safety (PR = 1.5; 95% CI = 1.1%–2.0%), Service (excluding Public Safety and Food) (PR = 1.3; 95% CI = 1.0%–1.7%) and Social Assistance/Healthcare (PR = 1.3; 95% CI = 1.1%–1.7%) industries. By employment situation, prevalence was significantly higher among self-employed respondents (PR = 1.3; 95% CI = 1.1%–1.6%) than among part-time paid employees (referent group).

**TABLE 2:** Weighted* prevalences and prevalence ratios of work-related health problems stratified by demographic and work characteristics — SummerStyles Survey, United States, 2018

Characteristic	Raw count (n = 2,425)^†^	Weighted % of work-related health problems (95% CI)	PR (95% CI)
**Sex**			
Men	1,307	36.7 (33.7–39.7)	1.1 (1.0–1.2)
Women	1,118	33.3 (30.2–36.4)	Referent
**Age group (yrs)**			
18–24	107	21.7 (13.8–29.6)	Referent
25–34	445	34.5 (29.7–39.3)	1.6 (1.2–2.0)
35–44	553	34.6 (30.2–38.9)	1.6 (1.2–2.0)
45–54	593	39.5 (35.1–43.9)	1.8 (1.4–2.3)
55–64	568	41.3 (37.0–45.6)	1.9 (1.5, 2.4)
65–74	139	33.0 (25.0–40.9)	1.5 (1.1–2.1)
≥75	20	29.1 (8.4–49.8)	1.3 (0.6–3.0)
**Race/Ethnicity**			
White, non-Hispanic	1,766	35.2 (32.7–37.6)	1.2 (1.0 1.6)
Black, non-Hispanic	218	39.9 (32.8–46.9)	1.4 (1.1–1.9)
Other, non-Hispanic	128	28.2 (19.8–36.5)	Referent
Hispanic	239	33.8 (27.2–40.4)	1.2 (0.9–1.6)
Multiracial, non-Hispanic	74	49.1 (36.6–61.6)	1.7 (1.1–2.6)
**Education**			
Less than high school	81	39.2 (27.4–51.0)	1.3 (1.0–1.6)
High school	558	38.7 (34.1–43.3)	1.3 (1.1–1.5)
Some college	682	37.3 (33.2–41.3)	1.2 (1.1–1.4)
Bachelor's degree or higher	1,104	30.6 (27.6–33.6)	Referent
**Employment situation**			
Full-time paid employee	1,814	35.3 (32.8–38.2)	1.1 (1.0–1.3)
Part-time paid employee	383	31.6 (26.4–36.8)	Referent
Self-employed	228	41.2 (33.9–48.4)	1.3 (1.1–1.6)
**Industry**			
Construction	89	48.6 (36.5–60.6)	1.6 (1.2–2.2)
Manufacturing	191	35.4 (27.6–43.2)	1.2 (0.9–1.6)
Wholesale or Retail Trade	196	35.1 (27.5–42.8)	1.2 (0.9–1.5)
Education	285	29.8 (23.9–35.7)	Referent
Food service	127	38.3 (28.7–47.9)	1.3 (1.0–1.7)
Public Safety	68	43.3 (30.1–56.6)	1.5 (1.1–2.0)
Service, excluding Public Safety or Food	278	38.9 (32.4–45.4)	1.3 (1.0–1.7)
Mining, Oil or Gas Extraction and Agriculture, Forestry, or Fishing	64	40.6 (25.9–55.2)	1.4 (1.0–1.9)
Transportation, Warehousing or Utilities	112	39.1 (28.7–49.5)	1.3 (1.0–1.8)
Other sector/None of the above	662	29.0 (25.1–32.9)	1.0 (0.8–1.2)
Social assistance and Healthcare	352	39.4 (33.7–45.2)	1.3 (1.1,1.7)
**Occupation**			
Manager	444	38.7 (33.5–43.8)	1.3 (1.0–1.6)
Professional	793	32.6 (28.9–36.3)	1.1 (0.8–1.3)
Community/Social Service	78	38.6 (26.0–51.3)	0.9 (0.7–1.0)
Services	358	38.2 (32.4–44.0)	1.2 (1.0–1.6)
Sales	181	30.8 (23.2–38.4)	Referent
Production and related	152	37.5 (28.9–46.1)	1.2 (0.9–1.6)
Other/None of the above	416	34.0 (28.8–39.2)	1.1 (0.9–1.4)
**Work arrangement**			
Independent contractor, independent consultant, or freelance worker	223	41.9 (34.6–49.2)	1.3 (1.0 1.8)
Paid by a temporary agency	35	37.5 (18.8–56.1)	1.2 (0.8–1.8)
Work for a contractor who provides workers and services to others under contract	77	39.1 (26.6–51.6)	1.2 (0.9–1.7)
Regular, permanent employee (standard work arrangement)	1,953	34.4 (32.1–36.8)	1.1 (0.8–1.4)
Some other work arrangement	134	31.8 (22.7–40.9)	Referent

**Abbreviations**: CI = confidence interval; PR = prevalence ratio.

* By gender, age, income, race, ethnicity, household size, education, U.S. Census region, and metro status, using U.S. Current Population Survey proportions.

^†^ Some categories do not sum to the total because of missing values.

## Discussion

A history of self-reported, work-related injury or illness is common in the working population; approximately one in three currently employed workers reported having experienced at least one health problem related to work during their lifetime. In this online panel survey, the prevalence of self-reported, work-related health problems varied by industry, employment situation, and certain demographic characteristics.

The current study provides the broadest published estimate of the total lifetime prevalence of occupational morbidity in the United States. This estimate is similar to findings from the 2005 European Working Conditions Survey, which estimated that an average of 35% of workers across 27 European Union countries reported that their work affected their health (*7*). An occupational health supplement to the 1988 National Health Interview Survey found that the overall prevalence of any of a set of 13 work-related chronic conditions was 7.5% among U.S. adults who had ever worked; however, that study did not include work-related injuries or acute illnesses and has not been repeated. Most studies focus on specific work-related health outcomes or exposures, not the overall prevalence of occupational morbidity (*8*). Available research on the overall occurrence of occupational morbidity typically estimates annualized incidence rates. The Bureau of Labor Statistics (BLS) reported an incidence rate of 2.8 cases per 100 full-time equivalent workers in 2018 (*1*). BLS estimates are based on employer reporting of certain types of injuries and illnesses. A 2019 study added to BLS estimates by combining additional resources to account for limitations in the BLS’s scope and incorporating attributable fractions to estimate additional types of work-related illnesses and injuries but was still limited to annual incidence estimates (*9*). The current study uniquely estimates lifetime (or cumulative) work-related morbidity and provides complementary industry and occupation-specific estimates of total nonfatal work-related health problems among currently working adults in the United States.

The findings in this report are subject to at least four limitations. First, the data are self-reported, so there is potential for recall and response bias. If a respondent developed a work-related health problem early in their employment, they might be less likely to recall a problem compared with a respondent who either recently experienced or received a diagnosis of a health problem. Depending on how respondents view the survey, they might also be more inclined or less inclined to report that they had a work-related health problem. Second, only those persons who were currently employed were included in the study, so the results could underestimate the prevalence of occupational health problems in the entire population. Third, variance might be underestimated because no sample design variables were available from SummerStyles. Finally, there were small numbers within certain groups such as workers paid by temporary agencies, resulting in very wide confidence intervals for estimates for these subgroups.

Occupational health surveillance relies on data from a variety of sources, including employer-based reporting, public health case reporting, workers’ compensation claims, health care records, and population-based surveys. All of these sources have limitations, and surveillance research is needed to determine how their use for occupational health surveillance can be improved (*10*). This is one of the few studies that estimates the lifetime prevalence of total work-related health problems and compares them among different industries. Although this study provides new information, more could be done to measure occupational morbidity. Studies using samples weighted specifically to be representative of industry and occupation groups and larger sample sizes are needed to more accurately represent the distribution of work-related health problems. Because respondents who left the workforce because of a work-related health problem, retirement, family commitments, or other reasons were not captured by this analysis, these results are still not capturing the entirety of work-related illnesses and injuries in the United States. Work-related health problems likely represent a public health problem that is larger than is assumed because of lack of information. Workplace prevention programs should be considered to decrease work-related health problems, especially in the higher prevalence industries of Construction, Public Safety, Service (excluding Public Safety and Food), and Social Assistance and Healthcare.

**Summary**

**What is already known about this topic?**

Workers are subject to injury and illness related to their work. Current surveillance methods likely underestimate the prevalence of occupational injuries and illnesses in the population.

**What is added by this report?**

A history of perceived work-related injury or illness is common among the working population (35.1%), and the prevalence varies by employment situation, industry of employment, and some demographic characteristics.

**What are the implications for public health practice?**

Workplace injury and illness prevention programs are needed to prevent work-related health problems, such as back pain, and reduce the number of health problems in higher-risk industries such as construction.

## Footnotes

* Weights were used to match the U.S. Current Population Survey proportions for gender, age, income, race, ethnicity, household size, education, U.S. Census region, and metro status.
